# A case study assessing the impact of mating frequency on the reproductive performance of the Hawaiian bobtail squid *Euprymna scolopes*

**DOI:** 10.1186/s42826-023-00168-1

**Published:** 2023-07-28

**Authors:** Andrew G. Cecere, Rachel A. Cook, Tim I. Miyashiro

**Affiliations:** 1 Department of Biochemistry and Molecular Biology, The Pennsylvania State University, University Park, PA USA; 2 The One Health Microbiome Center, Huck Institutes of the Life Sciences, The Pennsylvania State University, University Park, PA USA

**Keywords:** Symbiosis, *Euprymna scolopes*, *Vibrio fischeri*, Reproductive performance, Cephalopod

## Abstract

**Background:**

The symbiosis between the Hawaiian bobtail squid *Euprymna scolopes* and bacterium *Vibrio fischeri* serves as a model for investigating the molecular mechanisms that promote the initial formation of animal-bacterial symbioses. Research with this system frequently depends on freshly hatched *E. scolopes*, but the husbandry factors that promote hatchling production in a mariculture facility remain underreported. Here we report on the reproductive performance of *E. scolopes* in response to decreased mating frequency.

**Results:**

One animal cohort was maintained in a mariculture facility for 107 days, with females assigned to either a control group (mating once every 14 days) or an experimental group (mating once every 21 days). No differences between the groups were observed in survival, the number of egg clutches laid, or hatchling counts. Each group featured multiple females that were hyper-reproductive, *i.e.*, they generated more than 8 egg clutches while in captivity. Examination of the distributions for daily hatchling counts of individual egg clutches revealed significant variation in the hatching patterns among clutches that was independent of mating frequency. Finally, an assessment of hatchling production showed that 93.5% of total hatchlings produced by the cohort were derived from egg clutches laid within the first 70 days.

**Conclusions:**

These results suggest a lower mating frequency does not impede hatchling production. Furthermore, the variation in hatchling production among egg clutches provides new insight into the reproductive performance of *E. scolopes* as a lab animal for microbiology research.

**Supplementary Information:**

The online version contains supplementary material available at 10.1186/s42826-023-00168-1.

## Background

Animals often depend on beneficial bacteria for specific traits that promote normal host physiology, development, and behavior. Many, if not most, of these beneficial associations depend on the host acquiring the bacteria from an environmental reservoir each generation [1]. The molecular details underlying the transmission routes for most beneficial animal-bacterial associations are poorly understood but are important for developing strategies that promote their formation as a way to improve host health.

The sepiolid (bobtail) squid *Euprymna scolopes* hosts populations of the beneficial bacterium *Vibrio fischeri* within a symbiotic light organ, where they produce bioluminescence that enables the squid to avoid detection through an antipredatory behavior known as counterillumination [2, 3]. The association is considered a mutualistic symbiosis, due to the bacteria receiving host-derived nutrients and energy sources that facilitate growth in exchange for bioluminescence production [4–6]. Within hours after *E. scolopes* squid have hatched from their eggs, the hatchlings have acquired *V. fischeri* cells from the seawater environment, thereby initiating the formation of a lifelong association [7]. The ability to culture *E. scolopes* independently from *V. fischeri* has enabled researchers to conduct squid-colonization assays that have provided fundamental insights into the molecular factors that promote symbiotic associations among animals and bacteria [1, 8]. Thus, reports on *E. scolopes* husbandry are critical for sustaining and improving the ability of laboratories to produce hatchlings for research purposes [9, 10].

The mating process for *E. scolopes* represents an underexamined but important aspect of animal husbandry for producing hatchlings. For *E. scolopes*, copulation is similar to other Sepiolidae and occurs during mating when the male inserts its hectocotylized left arm into the mantle cavity of the female for the implantation of spermatophores into the female bursa copulatrix [11, 12]. After introducing partners into the same tank, mating events occur rapidly without obvious courtship behavior and last between 25 and 80 min [11, 13, 14]. After mating dissolution, the squid can be housed in separate tanks. Egg fertilization is not directly associated with the mating event but instead occurs when spermatophores are everted within the female mantle to release and activate the sperm near the eggs [14]. The ability of females to retain spermatophores has been suggested to enable sperm competition among males [11]. Prior to egg deposition, the eggs are also coated with layers of jelly that contain a consortium of microbes with antifungal properties that protect the eggs from fouling [15, 16]. The eggs, which are deposited as a clutch to the underside of hard substrates including plastic pipes, undergo embryonic development and hatch within 18–26 days [11, 17].

Here, we continue our effort to increase knowledge associated with the care and maintenance of *E. scolopes* for use in research. We approached this study with three questions in mind. First, we asked whether the frequency of mating in captivity affects hatchling production. Generally, there is consensus that certain phases of the mating event by Sepiolidae can incur damage to various tissues of the female, such as when the male grasps the female by multiple arm pairs to prevent escape [12], which raises the possibility of improving fitness of the female by lowering mating frequency. Second, because our previous study could not establish how many hatchlings each squid produces due to different clutches being maintained in the same chamber [10], we asked to what extent does an individual female contribute to the production of hatchlings? Finally, to gain insight into the hatching dynamics of egg clutches, we asked at what rate does each egg clutch produce hatchlings?

## Results

**Fig. 1:**
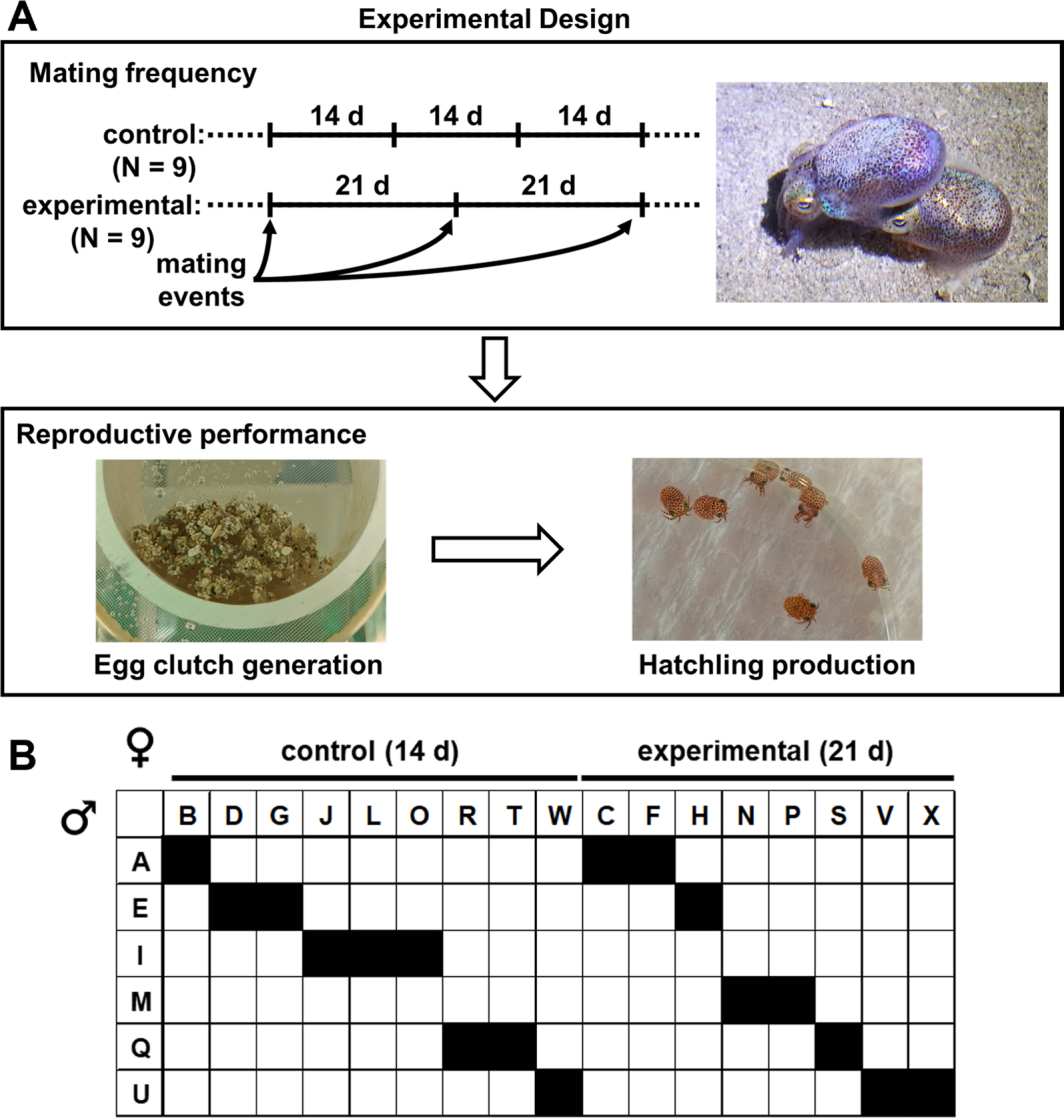
Experimental design. **A** *Top*, mating frequency was lowered to once every 21 days for experimental group relative to the conventional rate of once every 14 days (control). *Bottom*, reproductive performance of each female was assessed by tracking the generation of egg clutches and corresponding production of hatchlings. **B** Mating pairs established for each group

Maintaining a cohort of adult *E. scolopes* squid in a mariculture facility for the purpose of producing hatchlings for research depends on providing regular opportunities for female and male squid to mate [18]. In our previous report, we paired each female with a male once every 14 days [10], which for a cohort of 24 animals (18 females and 6 males) consists of each male mating with 3–4 females. Because females retain spermatophores that can be used for future egg fertilization events [14], we hypothesized that a lower mating frequency reduces stress on a female without affecting her reproductive performance. Therefore, to determine whether mating frequency affects the reproductive performance of *E. scolopes*, we lowered the frequency of pairing partners for half of the females (N = 9) to once every 21 days (experimental group) (Fig. 1). The remaining females of the cohort (N = 9) were maintained on a conventional mating schedule of 14 days (control group).

**Fig. 2:**
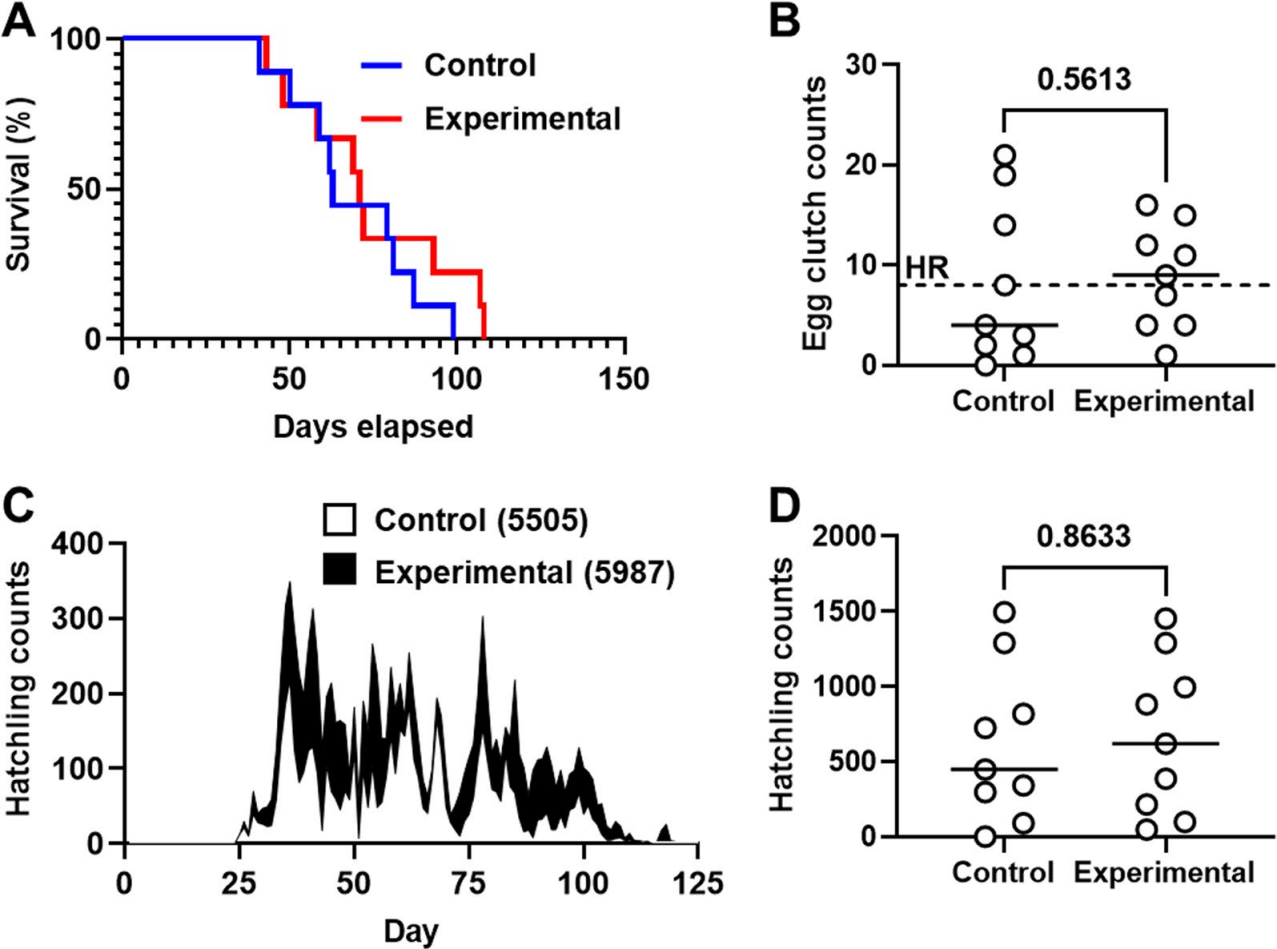
Reproductive performance of *E. scolopes*. **A** Survival curves of control (blue) and experimental (red) groups (N = 9 females per group). **B** Egg clutches generated by control and experimental groups, with each point representing an individual female. Dotted line indicates the cutoff (8 egg clutches) above which an animal is scored as hyper-reproductive (HR). *P* value calculated from Mann–Whitney test. **C** Stacked area chart with areas indicating hatchling counts from control (white) and experimental (black) groups. **D** Hatchlings produced by control and experimental groups, with each point representing an individual female. *P* value calculated from Mann–Whitney test

Overall, the experimental and control groups had 30 and 41 mating events, respectively. Each group exhibited similar survival curves, with the median only slightly longer for the experimental group (71 days) than the control group (63 days) (Fig. 2A, Mantel-Cox test, *p* value = 0.4221), which suggests that the lower mating frequency did not affect animal fitness. Both groups generated similar numbers of clutches (experimental = 79 and control = 71) (Fig. 2B), which produced comparable numbers of hatchlings (experimental = 5987 and control = 5505) (Figs. 2C, D). In addition, the frequency of hyper-reproductive animals, *i.e.*, animals that produce > 8 egg clutches [10], did not vary between groups in a statistically significant manner (experimental = 5/9 and control = 3/9; two-tailed two-proportional Z-test, *p*-value = 0.34212), which suggests that hyper-reproductivity was not altered by the change in mating frequency. Taken together, these results suggest that the lower mating frequency did not impact the reproductive performance of *E. scolopes* in a way that affects hatchling production by the mariculture facility.

**Fig. 3:**
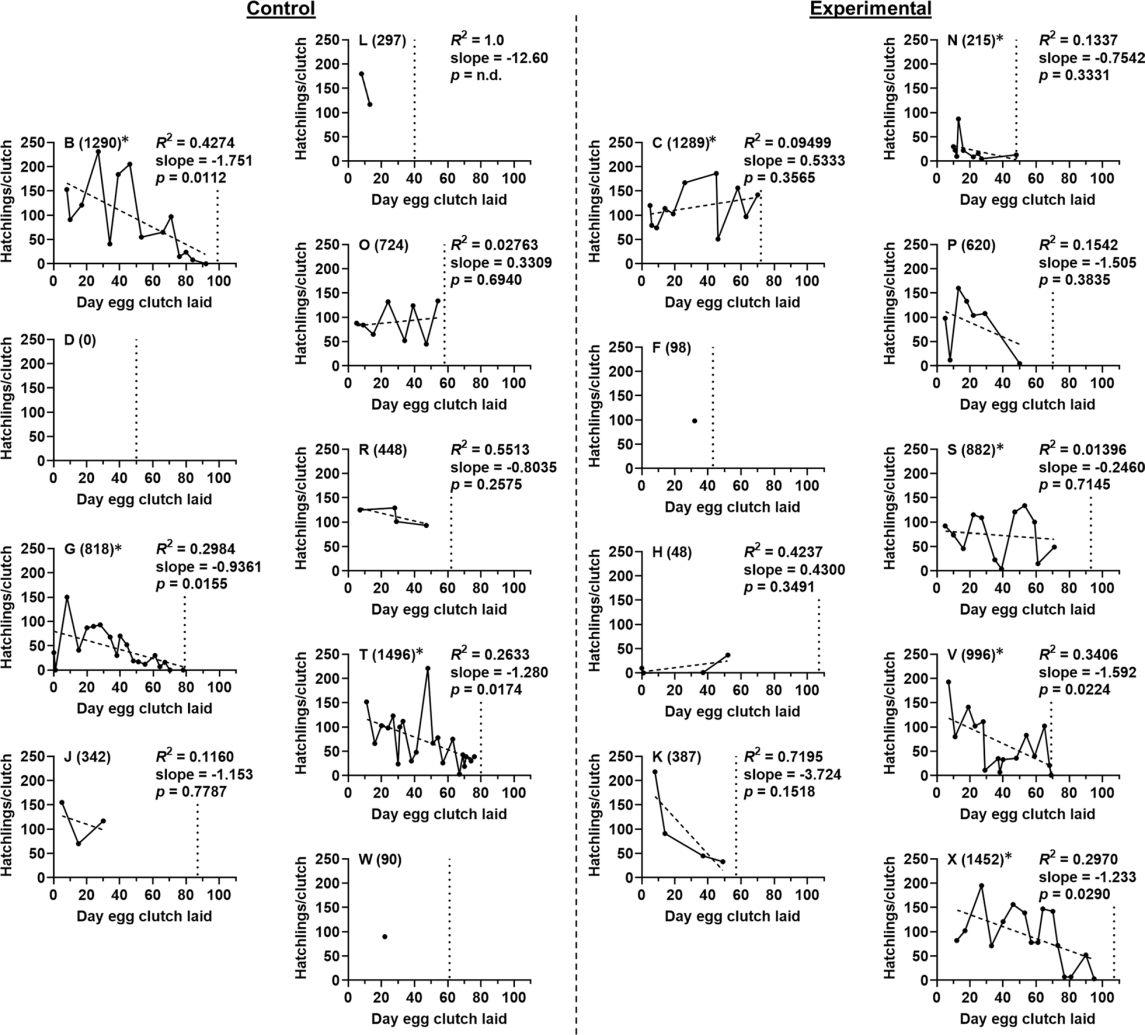
Hatchling production for individual *E. scolopes*. Each point represents the number of hatchlings produced by an egg clutch laid on the indicated day by one adult (labeled with letter). Total hatchlings shown in parentheses, and an asterisk indicates the squid was a hyper-reproductive animal. Vertical dotted line marks the mortality of the corresponding animal, with animals euthanized by day 107. The dashed line indicates the fit derived from linear regression of the data for animals that laid at least two egg clutches, with corresponding R-squared and slope displayed. An F test was performed to consider the reduced model with a slope of zero, with *p* value ≤ 0.05 rejecting this null hypothesis

In our previous report, some egg clutches were housed in the same chamber [10], which prevented an accurate count of the hatchlings produced by each egg clutch. In this study, each egg clutch was housed individually, which enabled tracking of both egg clutch generation and hatchling production by each female over time (Fig. 3). These data revealed a strong positive correlation between the number of egg clutches generated with the corresponding number of hatchlings produced (Table 1). A smaller positive correlation was found between the duration females were maintained in the facility (survival) and hatchlings that were produced (Table 1). In contrast, the number of hatchlings produced by each female was not correlated with the number of mating events (Table 1). Taken together, these results highlight the significance of generating egg clutches for a mariculture facility to produce hatchlings for research.

**Table 1:** Correlation analysis of hatchling production

Comparison	Spearman r^a^	95% confidence interval	*p* value^b^
Hatchlings versus Egg clutches	0.8774	0.6876 to 0.9550	< 0.0001
Hatchlings versus Survival	0.5328	0.07287 to 0.8058	0.0228
Hatchlings versus Matings	0.3610	− 0.1421 to 0.7158	0.1411

^a^Nonparametric Spearman correlation

^b^*p* value derived from calculating a two-tailed t-ratio test with randomly sampling a distribution that has no correlation between variables

**Fig. 4:**
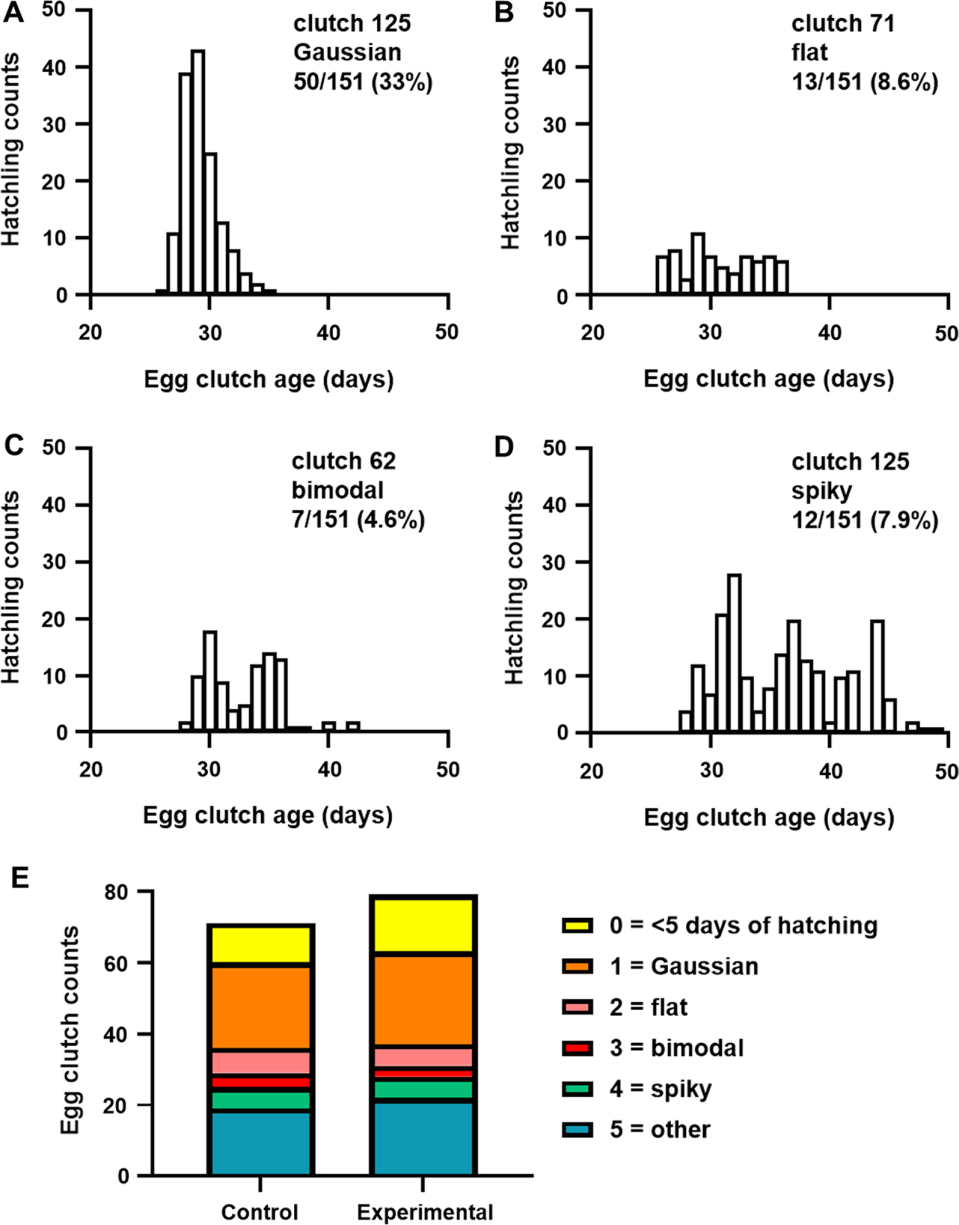
Daily hatchling production by egg clutches. **A**–**D** Example distributions with indicated shape and frequency among egg clutches of entire cohort. **E** Counts of egg clutches with indicated distributions among control and experimental groups

We also examined the production of hatchlings from each egg clutch over time. On average, egg clutches began hatching on day 26.4 ± 0.3 (relative to when the egg clutch was laid), with 50% of the hatchlings emerging by day 29.6 ± 0.4. The distributions of daily hatchlings varied among egg clutches, with only 33% (50/151) exhibiting a normal distribution curve (Fig. 4A). Other hatching patterns that were observed included distributions that were flat (Fig. 4B), bimodal (Fig. 4C), or spiky (Fig. 4D). Further examination of these distributions showed no effect by individual females nor mating frequency (Fig. 4E). Taken together, these data suggest that egg clutches can serve as a source of variation for the production of hatchlings.

**Fig. 5:**
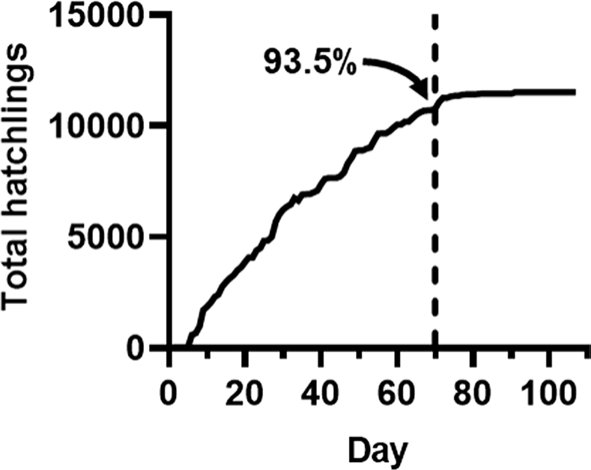
Hatchling production by animal cohort. Curve indicates the total number of hatchlings that would be produced by the egg clutches generated by the indicated cohort day. Dotted line indicates the median survival day of the cohort (70 days)

Maintaining a cohort of *E. scolopes* depends on staff effort and supplies, so the knowledge of how a mariculture facility functions over time can inform a cost–benefit analysis of its operation. The cohort described in this study ceased producing hatchlings on day 125, likely due to both female mortality and a decline in the number of hatchlings produced by egg clutches (Fig. 3). Consequently, the benefit (hatchling production) gained at the cost of maintaining the cohort decreased over time. Therefore, we asked how many hatchlings would be produced if the cohort was maintained for a shorter duration. In particular, euthanizing the animals remaining at the median survival date (day 70) resulted in the cohort still able to produce 10,747 hatchlings, *i.e.*, shortening the cohort maintenance time by 38 days (final animal was euthanized on day 108) would have only prevented the production of 745 hatchlings (Fig. 5).

## Discussion

The *E. scolopes*–*V. fischeri* symbiosis is an important experimental system for modeling how animals acquire bacterial symbionts from an environmental reservoir. Research in this field depends on the production of hatchlings within a laboratory setting. The role of mating frequency on hatchling production is an underexamined aspect of maintaining a cohort of *E. scolopes*. Therefore, this study was designed to assess whether mating frequency influenced hatchling production by *E. scolopes*. Overall, we found that decreasing the mating frequency from 14 to 21 days did not affect survival, egg clutch generation, or hatchling production (Fig. 2). Consequently, lowering the rate at which animals are paired for mating events can likely be implemented as one way to lower female stress and decrease the personnel effort associated with maintaining *E. scolopes* without compromising the hatchling production. Further investigation into the role of mating frequency on hatching production will require future studies that feature larger perturbations in the mating frequency and a negative control group comprising females that are not coupled with males.

A previous report highlighted that hatchlings emerge over several days [18], which enables each clutch to contribute to the total hatchling counts of a mariculture facility for multiple days. The data presented here reveals that there is in fact large variation in the distribution pattern of daily hatchlings among egg clutches (Fig. 4), but the underlying mechanism remains unclear. One intriguing possibility is that there is sperm competition for egg fertilization that arises due to the ability of female squid to store spermatophores [11]. Consequently, the eggs comprising a single clutch may have been fertilized by spermatophores acquired from different mating events, including those that occurred prior to the female being captured.

The results presented here reveal two primary factors that contribute to hatchling production by a cohort of *E. scolopes*. First, the number of hatchlings produced by a female is strongly correlated with the number of egg clutches that it laid (Table 1). Consequently, future studies of hyper-reproductivity, which is observed in approximately half of the animals in each cohort [10], will likely inform husbandry techniques that could boost hatchling production and potentially reduce the number of wild-caught animals needed for research [19]. Second, we observed that hatchling production decreases for females that survive beyond day 70, due to fewer egg clutches being laid or to fewer hatchlings resulting from each clutch over time (Fig. 3). This overall decline in daily hatchling production results in the precipitous drop in hatchling counts after day 100 (Fig. 2C).

Knowledge of the reproductive performance of *E. scolopes* in captivity has important practical implications for maintaining a mariculture facility. Because most squid colonization assays require the sample size to exceed five animals per group, it becomes difficult to perform experiments with the lower daily hatchling counts associated with an older animal cohort. The analysis in this study represents an important step in better defining the parameters for maintaining a cohort of *E. scolopes* for research purposes. Future studies will expand on this analysis to provide forecasting models for researchers in this field to determine appropriate cohort sizes for specific hatchling counts.

## Conclusions

This report assessed the reproductive performance of *E. scolopes*. Lowering the mating frequency from once every 14 days to once every 21 days did not affect egg clutch generation or hatchling production. Egg clutches displayed a variety of patterns in daily hatching production. The knowledge of how individual *E. scolopes* squid contribute to hatchling production informs animal husbandry techniques, which improves the use of *E. scolopes* as a lab animal for microbiology research.

## Methods

### Collection, transport, and acclimation

Mature *E. scolopes* squid were gathered at Maunalua Bay, Oahu, HI from March 4–7, 2022 and maintained as described previously [10]. Following the established animal shipment methodology [10], two crates of squid (each containing 10 females and 3 males) were shipped from Daniel K. Inouye International Airport (Honolulu, HI) to Pennsylvania State University (University Park, PA) via United Parcel Service (UPS). Both crates were shipped simultaneously on March 7, 2022 and arrived together the following day after approximately 18 h in transit. On arrival, animals were assessed by evaluating color and responsiveness, and water quality of shipping bags was tested using established colorimetric tests [10]. All animals were acclimated and maintained following procedures laid out in previous work [10].

### Mating

Following a brief acclimation period, female squid were separated at random into two groups distributed evenly throughout the adult tanks. Care was taken to ensure that both groups contained a similar range of animal sizes. In one group (control) females were mated with an individual male at the previously defined standard of once every 14 days [10]. In the other group (experimental), mating events were once every 21 days. Once a successful mating had occurred, the mating pair was conserved for the duration of the experiment. Each female continued to mate at the defined frequency for the remainder of its survival.

### Egg clutches/hatchlings

Adult tanks were examined daily for freshly laid egg clutches within a PVC cave. If discovered, egg clutches were individually transferred to a separate egg system within labeled hatchery chambers where they were removed from their PVC cave and documented. Each clutch would then remain untouched in isolation during an incubation period of ~ 20–30 days. Clutches were monitored twice daily for hatchlings. When encountered, hatchlings from each clutch were counted and recorded during collection for experimental use.

### Data acquisition and analysis

Data were collected, organized, and analyzed using Microsoft Excel. GraphPad Prism v. 9.3.1 (GraphPad Software, LLC) was used to generate graphs, perform linear regression equations, and test for statistical significance between groups.

### Analysis of hatchling production by egg clutches

The distribution of daily hatchling counts for each egg clutch was graphed, and its shape was visually scored using the categories: 0 =  < 5 days of hatchlings, 1 = Gaussian, 2 = flat, 3 = bimodal, 4 = spiky, and 5 = other. Each distribution was scored independently by three researchers, and the mode of the scores was reported. For cases of three different scores, those distributions were scored as 5 (other).

## Supplementary Information

**Additional file 1.** Excel workbook with data for Figures 2, 3, 4, and 5.

## Data Availability

All data are provided in the accompanying supplemental data file [see Additional file 1].
